# Immobilization of halophilic *Aspergillus awamori* EM66 exochitinase on grafted k-carrageenan-alginate beads

**DOI:** 10.1007/s13205-015-0333-2

**Published:** 2016-01-11

**Authors:** Mona A. Esawy, Ghada E. A. Awad, Walaa A. Abdel Wahab, Magdy M. M. Elnashar, Ahmed El-Diwany, Saadia M. H. Easa, Fawkia M. El-beih

**Affiliations:** 1 Department of Chemistry of Microbial and Natural Products, National Research Centre, Tahrir Street, Dokki, Cairo, Egypt; 2 Department of Microbiology, Faculty of Science, Ain Shams University, Khalifa El-Maamon St., Cairo, Egypt; 3 Centre of Scientific Excellence-Group of Encapsulation and Nanobiotechnology, Polymers Department, National Research Center, El-Behooth St., Dokki, Cairo, Egypt; 4 Biomedical Science Department, Health Science School, Curtin University, Bentley, WA 6102 Australia

**Keywords:** Exochitinase, Honey isolate, *Aspergillus awamori*, Immobilization

## Abstract

A novel extreme halophilic exochitinase enzyme was produced by honey isolate *Aspergillus awamori* EM66. The enzym*e* was immobilized successfully on k-carrageenan-alginate gel carrier (CA) with 93 % immobilization yield. The immobilization process significantly improved the enzyme specific activity 2.6-fold compared to the free form. The significant factors influencing the immobilization process such as enzyme protein concentration and loading time were studied. Distinguishable characteristics of optimum pH and temperature, stability at different temperatures and NaCl tolerance for free and immobilized enzyme were studied. The immobilization process improved optimum temperature from 35 to 45 °C. The immobilized enzyme retained 76.70 % of its activity after 2 h at 75 °C compared to complete loss of activity for the free enzyme. The reusability test proved the durability of the CA gel beads for 28 cycles without losing its activity.

## Introduction

Osmophilic microorganisms survive environmental extremes of desiccation, pressure and acidity; so it is expected that their bio-products will have some unique properties to adapt to such extreme conditions. In recent years, significant progress has been made in discovering and developing novel isolates from honey since it contained a great variety of dominant spores which are expected to have unique feature (Esawy et al. 2011, 2013). This expectation comes from the osmophilic nature of honey and its constituents which are mainly fructose (about 38.5 %) and glucose (about 31.0 %), Crosby (2004).

Chitinases can be divided into two major categories endochitinases and exochitinases (Graham and Sticklen 1994). Exochitinases can be divided into two subcategories: exochitinases and β-1, 4 *N*-acetyl glucosaminidases. Exochitinases (EC 3.2.1.29), the objective of this study, catalyze the progressive release of diacetylchitobiose starting at the non-reducing end of chitin chains (Chuan 2006).

Several chitinolytic enzymes have been identified in various fungi including, *Trichoderma harzian* (El-Katatny et al. 2000; Sandhya et al. 2004) *Trichoderma* *longibrachiatum* IMI 92027 (Kovacs et al. 2004) *Aspergillus niger* LOCK 62 (Brzezinska and Jankiewicz 2012) and *Aspergillus terreus* (Ghanem et al. 2010).

Chitinolytic enzymes have been widely used in various processes including agricultural, biological and environmental fields (Graham and Sticklen 1994). Chitinases have received attention due to their use as a biocontrol agent (Mathivanan et al.^1998^) and developing transgenic plants (Wang et al. 2008).

Halophilic enzymes, known as extremozymes produced by halophilic microorganisms, have identical enzymatic features like their non-halophilic counterpart, but they exhibit different properties mainly in structure. Among these, two main points could be mentioned, (a) a high content in acidic amino acids located predominantly at the protein surface and (b) requirement for high salt concentration for better biological functions. The halophilic chitinases are quite important in view of the accumulation of a huge amount of chitin, the second most abundant renewable biomass after cellulose, particularly as arthropod integuments in marine environments (Elnashar et al. 2009).

Chitinase immobilization is necessary for hydrolysis of oligosaccharides and the reusability of the enzyme. Searching for carriers suitable for enzyme immobilization with high efficiency, could be easily used in industries and relatively low cost is always the aim of many recent studies. The immobilization technique would enable the reusability of enzymes for tens of times. In addition, it reduces the enzyme cost significantly (Elnashar et al. 2009). There are many techniques to immobilize enzymes, such as adsorption, covalent, encapsulation, entrapment, and cross linking (Salman et al. 2008). Each technique has its pros and cons; however, covalent technique has the advantage of keeping the enzymes well bound to the carrier, avoiding enzyme diffusion, and this is why it is widely preferred on the industrial scale (Danial et al. 2010). Enzyme immobilization by covalent bonding to an insoluble polymer has the supposed advantage of irreversible binding of the enzyme to the support matrix (Trevan 1988).

Covalent bonds are stronger due to the constant sharing of electrons between atoms. Biopolymers, such as alginates, carrageenans, and chitosans, are commercially available, have diverse features, and are available at a reasonable cost, which could make them good candidates for immobilizing enzyme (Hugerth et al.^1997^). Recently, various micro carriers, have become available for enzyme immobilization (Wei et al. 2000; Jia et al. 2002; Lee et al. 2004; Sawicka et al. 2005). Typically, smaller particles provide a larger surface area for enzymes attachment and a shorter diffusion path for the substrates (Kim et al. 2005).

This investigation concerned with the *A. awamori* EM66 exochitinase immobilization onto k-carrageenan-alginate gel beads (CA). The factors influencing the immobilization yield were studied. The properties of the free and immobilized enzyme were investigated. The results indicated that, the high activity, thermo-tolerant and high salt-tolerant exochitinase produced by *A. awamori* EM66 could be useful for application in diverse areas such as biotechnology and agro-industry.

## Materials and methods

### Chemicals

k-Carrageenan (Mr: 154,000; sulfate ester ~25 %), alginic acid sodium salt from brown algae (CAS # 9005-38-3), Chitin from crab shells and *p*-nitrophenyl-β-d-*N*-acetyl glucoseaminide (PNP-β-GlcNAc) were purchased from Fluka Biochemika Co. (USA). Polyethyleneimine (MW: 423), Cat # 468533, was obtained from Aldrich. All other reagents were of the purest grade commercially available.

### Microorganism (isolation and identification)

The fungal used throughout this work was previously isolated from mountain honey bee collecting nectar from desert flower. Honey samples are fresh non-treated ripe honey (directly collected in beehives). It was identified based on morphological characterization and 18S rRNA sequence analysis (data not published yet). It was designed as *A. awamori* EM66. Gene Bank database was achieved in BLASTN searches at the National Center for Biotechnology Information (NCBI) site (http://www.ncbi.nlm.nih.gov) and take an Accs. NO (ACC No. KF774180).

### Media

#### Maintenance medium

The organism was maintained on the usual potato dextrose agar medium (PDA) preferable for fungal growth.

#### Fermentation medium

The optimized medium of exochitinase production contains % (glucose 0.4; soyabean 0.4; chitin 1.0; wheat flour 0.4; sodium nitrate 0.02; cobber sulfate anhydrous 0.001; magnesium sulfate 0.05; dipotassium hydrogen phosphate 0.2; ferric chloride 0.001; manganese sulfate 0.002; calcium chloride 0.05). The pH was adjusted to 8.0.

### Enzyme assay

Exochitinase activity was determined according to the method of (Matsumoto et al. 2004) using the chromogenic substrate *p*-nitrophenyl-β-d-*N*-acetyl glucoseaminide (PNP-β-GlcNAc) as a substrate. One unit of the enzyme activity was defined as the amount of enzyme releasing 1 µmol of *p*-nitrophenol per minute under the specified assay conditions.

### Protein content

This was estimated by the method of (Lowry et al. 1951) using Folin-Ciocalteu phenol reagent (FCR) and extrapolated from the standard curve of bovine serum albumin.

### Partial purification

The crude *A. awamori* EM66 exochitinase was fractionated using ethanol concentrations (30–80 %). The precipitate was obtained by centrifugation (10,000×*g*, 15 min at 4 °C) and suspended in an appropriate volume of 0.05 M acetate buffer (pH 5.0).

### Immobilization

The partially purified enzyme has been covalently immobilized onto alginate-carrageenan gel beads where half gram of gel beads was soaked for 24 h.

$${\text{Immobilization yield}} = \left( {I/A - B} \right)\left( \% \right)$$

*A* is the enzyme added (mU/carrier), *B* is the unbound enzyme (mU/carrier) and *I* is the immobilized enzyme (mU/carrier).

#### Preparation of grafted alginate-carrageenan beads

One g of alginate and 1 g of k-carrageenan were dissolved in distilled water to give a final concentration of 2 % (w/v) was dropped through 300 μm nozzle in a hardening solution using the two-step method (Danial et al. 2010) the beads were treated by 2 % (w/v) CaCl_2_ (Ca^2+^) for 2 h then were dropped in a solution of 2 % (w/v) CaCl_2_ dissolved in 4 % (v/v) polyethylenimine at pH 8 for 2 h. The treated gels beads were then soaked in a solution of 2.5 % (v/v) glutaraldehyde (GA) for 2 h to incorporate the new functionality, aldehyde group. The solutions were mixed thoroughly using an overhead mechanical stirrer until complete dispersion had occurred. The gels were prepared into uniform beads using the Inotech Encapsulator (Elnashar et al. 2013).

##### Immobilization of exochitinase onto grafted k-carrageenan-alginate beads and soluble protein determination

The k-carrageenan-alginate gel carrier (CA) was used to immobilize exochitinase by soaking 0.5 g of the gel beads in 2.5 mL of diluted enzyme contained 0.256 mg protein (appropriate dilution was done) in 0.05 M acetate buffer at pH 5.0 for 24 h. The beads were washed twice thoroughly for 30 min with acetate buffer to get rid of any unbound enzyme. The gel beads containing the immobilized enzyme were stored at 4 °C for further measurements. The supernatant and the wash were kept for soluble protein assay via bovine serum albumin (BSA) as a standard protein. Protein concentration of free enzyme was determined as described by (Lowry et al. 1951). The protein concentration of the immobilized enzyme was estimated by taking into consideration the protein concentration in the initial solution and of the unbound protein. The amount of protein immobilized onto and into the gel carrier Pg (mg/g) was calculated using the following equation:

$${\text{Pg }} = \frac{{C_{\text{o}} V_{\text{o}} - \, C_{\text{f}} V_{\text{f}} }}{W}$$

where *C*_o_ is the initial protein concentration (mg/mL), *C*_f_ the protein concentration of the filtrate (mg/mL), *V*_o_ the initial volume of the enzyme solution (mL), *V*_f_ the volume of filtrate (mL), and w is the weight of gel carrier used (g). The enzyme activity for immobilized enzyme was determined. An equal amount of enzyme (0.5 ml enzyme equivalent to 0.5 g gel of gel beads) and 0.5 ml of *p*-nitrophenyl-β-*N*-acetyl glucosaminide as substrate were incubated for 1 h at 30 °C. The reaction was stopped by addition of 25 ml of 0.125 M sodium borate buffer, (pH 10.1). The amount of released *p*-nitrophenol was measured at the absorbance 410 nm as mentioned previously.

### Effect of different incubation time on the activity of both partially purified and immobilized enzymes

In this experiment, the assay was done for different time periods ranging from 2 to 120 min to investigate the optimal incubation time at which maximum enzyme activity occurred. Reaction mixture contain equal amount of substrate dissolved in sodium acetate buffer at pH 5.0 and the enzyme as usual.

### Optimization of the immobilization yield (%)

#### Optimization of loading time

For determination of the optimum loading time, enzyme in suitable concentration was incubated with gel beads for different periods of time ranged from 2 to 24 h. After loading time was finished, the gel beads were washed twice thoroughly for 30 min with acetate buffer to get rid of any unbound enzyme and the usual assay has been implicated.

#### Optimization of the enzyme loading capacity

In this experiment, 0.5 g of the gel beads was soaked in 2.5 mL of original enzyme solution in addition to the different enzyme dilutions 1:2, 1:4, 1:6, and 1:8 in 0.05 M acetate buffer at pH 5.0 for 18 h. The gel beads were then washed twice thoroughly for 30 min with acetate buffer to get rid of any unbound enzyme and the usual assay has been implicated.

### Evaluation of exochitinase catalytic activity

Different characters of *A. awamori* EM66 exochitinase for free and immobilized form, like effect of reaction conditions (temperature, pH and substrate concentration), thermal stability and NaCl concentrations have been tested.

#### Operation temperature and pH profile

The optimal temperature was determined by incubating the reaction mixture at different temperatures from 25 to 60 °C. The optimal pH was investigated by measuring the activity between pH 4 and 7 using sodium acetate buffer (pH 4–5) and sodium phosphate (pH 5.5–7).

#### Thermal stability

Thermal stability has been achieved by incubating the partially purified and immobilized enzyme at different temperatures from (35 to 75 °C) at different time periods (30, 60, 90 and 120 min.). At the end of the incubation time, the usual enzyme assay has been carried out at the optimum conditions for each of the partially purified and immobilized enzyme.

#### *K*_m_ and *V*_max_ values of free and immobilized *A. awamori* exochitinase

The LineWeaver–Burk plot (double reciprocal) method, was used to obtain the Michaelis–Menten kinetic models adequate for the description of the hydrolysis of *p*-nitrophenyl-β-*N*-acetyl glucosaminide by the free and the immobilized enzymes, apparent *K*_m_ and *V*_max_ of free and immobilized exochitinase. The assay mixture comprised 0.84 and 0.178 mg/mL of free and immobilized enzyme, a substrate concentration of 0.25–4.0 mg at pH 5.0, assay temperature was 30 and 45 °C for free and immobilized enzymes, respectively.

#### Effect of different NaCl concentrations

One ml of enzyme contained 0.84 and 0.178 mg/mL of free and immobilized enzymes, respectively, was incubated with 1 ml of NaCl to reach final concentration ranged from (0.125 to 6 M) in the absence of substrate for half an hour, after that the usual enzyme assay has been carried out at the optimum conditions for each of the partially purified and immobilized enzymes.

### Reusability of immobilized exochitinase

Immobilized *A. awamori* EM66 exochitinase beads 0.5 g were incubated with 0.5 mL of *p*-nitrophenyl-β-*N*-acetyl glucosaminide as substrate for 10 min at 45 °C. The reaction was decanted and stopped by separating the gel beads from the substrate. Then they were washed with acetate buffer pH 5.0 and the first step was repeated to start a new reaction. The amount of released *p*-nitrophenol was measured at the absorbance 410 nm as mentioned previously.

### Storage stability

Both the immobilized and free exochitinase enzymes were stored in distilled water at 4 °C for 6 months. The activity was measured every 2 weeks using 0.84 and 0.178 mg of protein for free and immobilized enzyme, respectively.

### Statistical analysis

Data analysis was carried out with MICROSOFT EXCEL (2007). All data are presented as mean ± standard error of means. All results were independently replicated three times (*n* = 3), with three measurements per replicate. The mean of the repeated measurements yielded the value for each replicate.

## Results and discussion

### Partial purification

Microorganisms isolated from an extreme environment have harsh and challenging conditions expected to be characteristic by unique features. The osmophilic nature of honey recommended it to be a good source for dormant spores which have new properties (Esawy et al. 2013). Within this context, *A. awamori* EM66 which was previously isolated from mountain honey reported good exochitinase productivity. Under the optimized medium the enzyme productivity was 5998.91 mU/mL. The crude enzyme was partially purified using ethanol fractionation (30–80 %) v/v. Protein precipitated due to ethanol fractionation at 30 % v/v showed about 18 times purification and recovered 22 % of activity (in comparison to the overall activity of the culture supernatant). The partially purified enzyme was assayed for its total activity and protein content, which were found to be 23.34 U and 0.837 mg, respectively. Accordingly, the specific activity was calculated to be 27.89 U/mg. The high specific activity of this enzyme gave it tremendous interest; since it referred that it approaches to purity.

### Enzyme immobilization onto carrageenan-alginate gel beads (CA)

The partially purified enzyme was immobilized by covalent binding with 93 % immobilization yield (Table 1). The specific activity calculated on a bound-protein basis was 71.238 U/mg. This result indicated that the immobilization process increased the specific activity 2.6-fold higher than that of free enzyme. In this finding, Riordan et al. (1989) reported that co-immobilization of *Micromonospora chalcae* with chitin in calcium alginate resulted in twofold increase in chitinase activity compared to the free form. On the other hand, it was found that the activity of immobilized chitinase in the rotational magnetic field rise compared to those in the absence of it (Mizuki et al. 2013). Finally, it was reported in active *Serratia marcescens* chitinase immobilized successfully by covalent binding to the polymer (hydroxypropyl methyl cellulose acetate succinate, AS-L) Chen and Chang (1994).

**Table 1:** Immobilization of exochitinase using alginate/k-carrageenan carrier by covalent bond

Carrier used	Enzyme added (mU/carrier)	Unbound enzyme (mU/carrier)	Immobilized enzyme (mU/carrier)	Immobilization yield (%)
	(A)	(B)	(I)	(I/A−B)
Alginate/k-carrageenan	2643.74	1327.801	1227.767	93.33 ± 0.5

### Optimum incubation period

Investigation of the convenient time for the maximum enzyme activity is important for getting the maximum substrate conversion in a short time. The optimum incubation time for both forms of *A. awamori* EM66 exochitinase had been recorded as 10 min (data not shown).

### Optimization of the immobilization yield

**Fig. 1:**
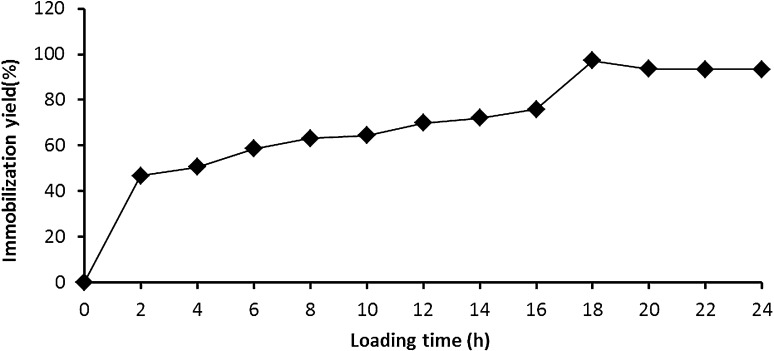
Optimization of the enzyme loading time expressed in immobilization yield (%) using grafted alginate/k-carrageenan prepared by covalent bond

**Fig. 2:**
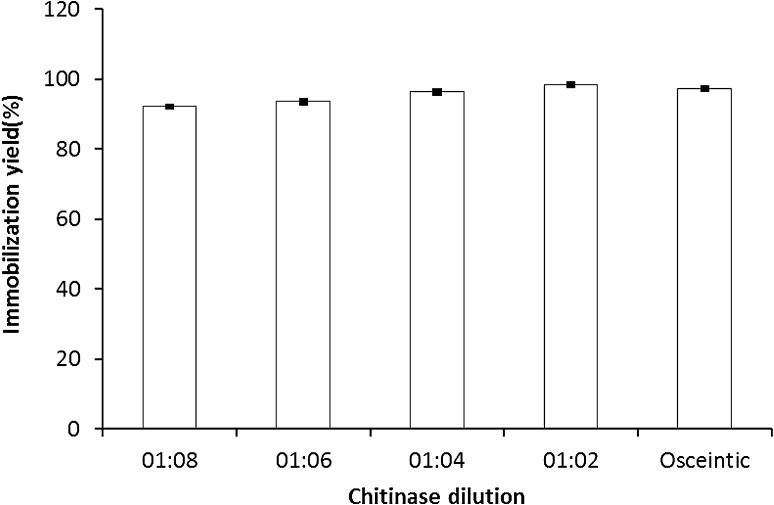
Optimization of the enzyme loading capacity expressed in immobilization yield (%) using alginate/k-carrageenan beads prepared by covalent bond

In these experiments, the conditions to get the maximum enzyme loading capacity (ELC) and time were studied. The result was expressed as immobilization yield (%). The immobilization yield increased proportionally with loading time reaching its maximum at 18 h incubation (97 %) (Fig. 1). The effect of different enzyme protein showed that *A. awamori*. EM66 exochitinase retained its complete enzyme activity at 1:2 dilutions; accordingly, the immobilization yield was 100 % as shown in Fig. 2. It is worthy to know that the obtained immobilization yield (100 %) is higher than the yield obtained by (Sakai et al. 1991).

### Temperature and pH

**Fig. 3:**
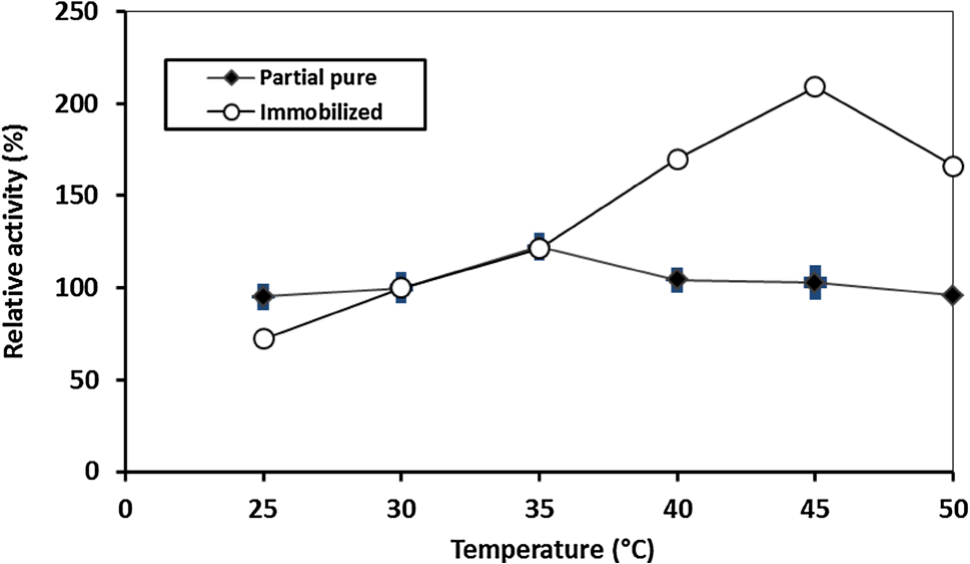
Optimum temperature profile of free and immobilized *A. awamori* ESAWY exochitinase

The optimal temperature was 35 and 45 °C for free and immobilized enzyme, respectively (Fig. 3.). The shift of the enzyme’s optimum temperature after immobilization could be regarded to the formation of a molecular cage around the protein (enzyme), which protected the enzyme’s molecules from the bulk temperature (Roger et al. 2004). On contrary, Sakai et al. (1991) reported that the optimum temperature of the immobilized *Nocardia orientalis* IFO 12806 chitinase decreased from 60 to 40–50 °C in free and immobilized form, respectively. The optimum pH for both partially purified and immobilized enzyme was 5.0 using acetate buffers (0.05 M) (data not shown). In this finding, Wang and Chio (1998) reported in the immobilization of *Pseudomonas aeruginosa* K-187 chitinase where the optimum pH and temperature shifted to pH 8 and 50 °C.

### Thermal stability

**Fig. 4:**
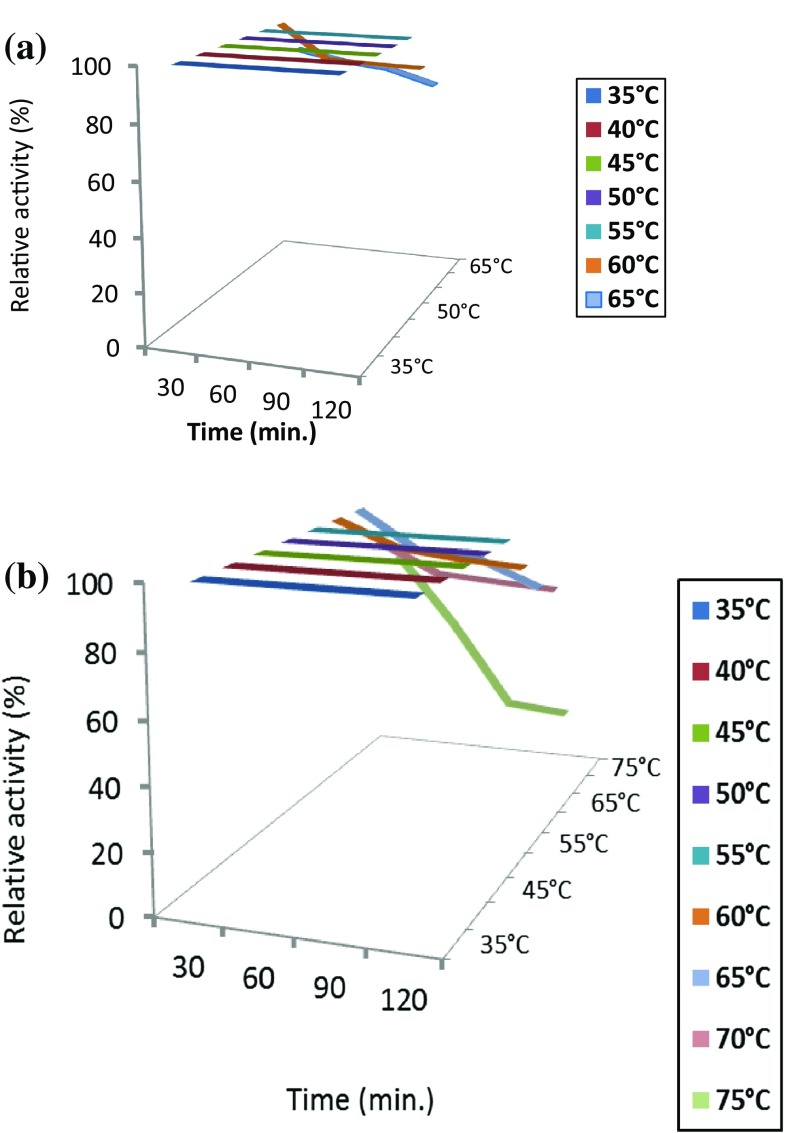
Thermal stability profile of free (**a**) and immobilized (**b**) *Aspergillus awamori* ESAWY exochitinase

The thermal stability for the free and the immobilized enzyme was evaluated. The results referred to the complete loss of free enzyme activity at (70 and 75 °C) while the immobilized retained about 71 % of its activity at 70 °C for 120 min and still active for about half activity at 75 °C for 1 h (Fig. 4a, b). Prasad and Palanivelu (2013) found that the recombinant thermostable *Thermomyces lanuginosus* immobilized chitinase showed remarkable thermo stability at 50 °C by retaining about 45 % of the activity for more than 6 h. It was reported that the *Bacillus* *thuringiensis* chitinase had three times increase in the stability compared to the free form (Escudero-Abarca et al. 2002).

### *K*_m_ and *V*_max_ of free and immobilized exochitinase

**Fig. 5:**
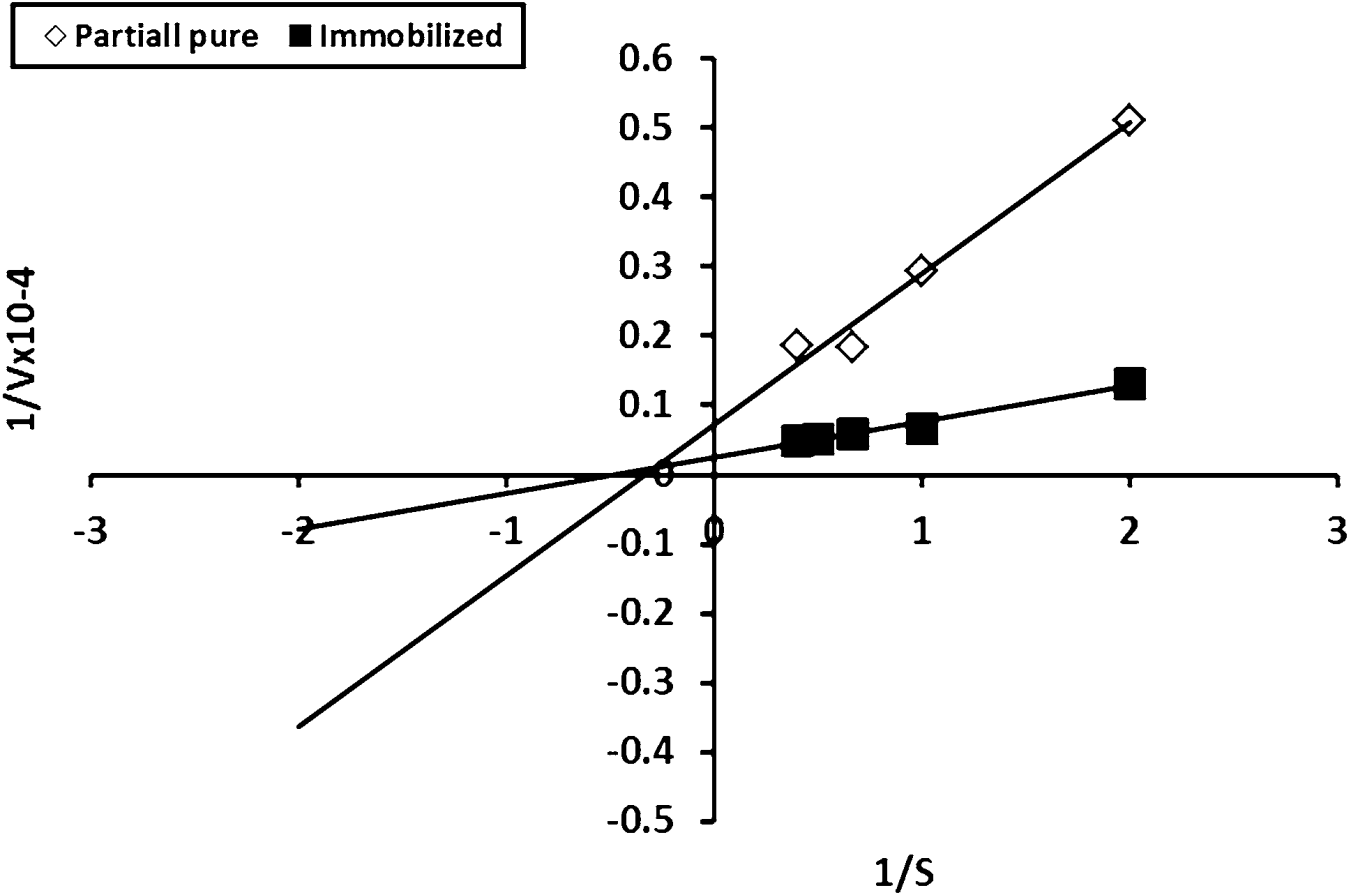
Kinetic constants of free and immobilized *A. awamori* exochitinase using Lineweaver–Burk plot method

The kinetic constants of free and immobilized exochitinase were calculated using the double reciprocal plot method (Lineweaver–Burk plot) as shown in Fig. 5 Different reaction mixtures had been prepared with different substrate concentrations to determine the enzyme kinetics (*K*_m_ and *V*_max_). Maximum activity occurred at 2.0 and 1.5 mg concentration for partial pure and immobilized enzyme, respectively. *K*_m_ was calculated as 0.33 and 2.0 mg/mL and *V*_max_ as 13.33 and 40.0 U/mg/min for free and immobilized form, respectively. The *K*_m_ value indicated that the partial pure enzyme is more sensitive to the substrate than the immobilized whereas the *V*_max_ value showed that the immobilized form is faster by about three times than the free form. It is worthy to mention that the kinetics of *A. awamori*. EM66 exochitinase differed from that of (Han et al. 2000) who recorded that acidic chitinases from the gizzards of a broiler had *K*_m_ and *V*_max_ of 0.42 mg/mL and 92.3 mg/mg protein/h, respectively, when the pentamer and hexamer of *N-*acetylglucosamine (GlcNAc) were used as a substrate, Moreover, the *K*_m_ and *V*_max_ values for *Serratia marcescens* B4A chitinase were 8.3 mg/mL and 2.4 m mol/min, respectively, as reported by (Zarei et al. 2011).

### Effect of different NaCl concentrations

**Fig. 6:**
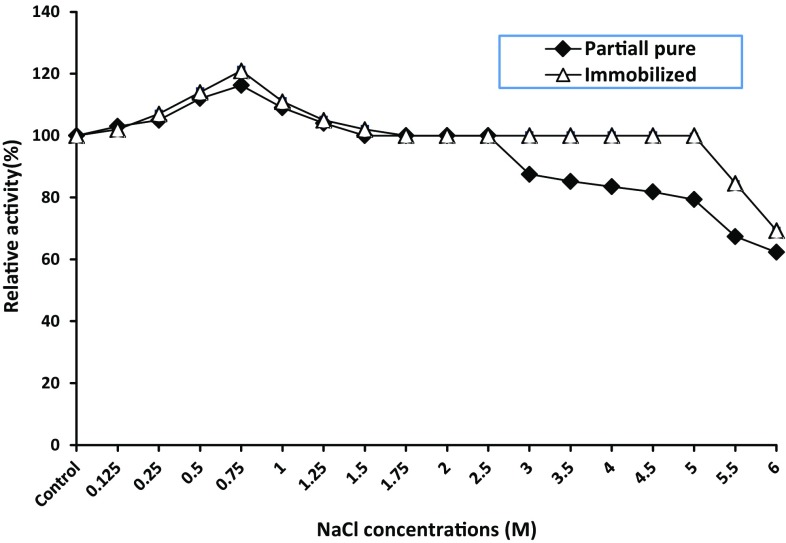
Evaluation of different NaCl concentrations on free and immobilized *Aspergillus awamori* ESAWY exochitinase

The effect of different NaCl concentrations on the free and immobilized enzyme was achieved (Fig. 6). The highest relative activity (116 and 120 % free and immobilized enzymes, respectively) was achieved at 0.75 M. In addition, both enzymes could tolerate the increase of NaCl concentrations to 6 M. This result could be attributed to the ability of honey microorganisms to adapt the honey osmophilic nature. Similar result was obtained from the honey isolate *Bacillus subtilis* NRC-B233b, where the dextranase activity increased about fourfold in the presence of 10 % NaCl (Esawy et al. 2012).

### Enzyme reusability

**Fig. 7:**
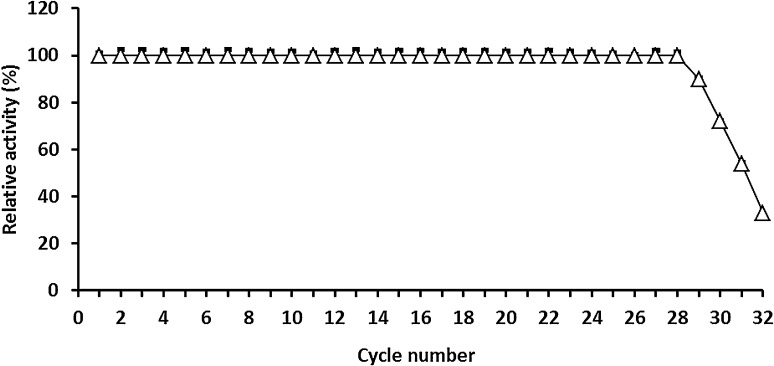
Repeated cycle of immobilized *Aspergillus awamori* ESAWY exochitinase

One of the most important targets, of immobilization was the enzyme ability to be reused efficiently for several times. The reusability of immobilized *A. awamori* EM66 exochitinase has been studied because of its importance of repeated use in the batch or continuous processes. The results indicated to the great ability of the immobilized enzyme to keep its complete activity after 28 cycles (Fig. 7). In this finding, Wang, and Chio (1998) found that the immobilized *Pseudomonas aeruginosa* k-187 chitinase retained 70 % of its original activity after 10 batches.

### Storage stability

Enzyme stability issues are always of high significance in the production of stable and reproducible biocatalysts (Danial et al. 2010). The storage stability is considered as an important parameter to confirm the enzyme stability. The results referred to the great stability of the free and immobilized form, both forms keep their complete stability at 4 °C for more than 6 months.

## Conclusion

Till now there are no sufficient studies concerning exochitinase immobilization, accordingly our main purpose in this research aimed to characterize the free and immobilized enzyme from the novel honey isolate *A. awamori* EM66. In general, the immobilization process improved the enzyme properties such as temperature and thermal stability. In addition, the results referred to the thermo-stability character of both free and immobilized *A. awamori* EM66 chitinase, in addition to its unique halophilic property. The free enzyme showed good activity against *Fusarium oxysporum* which causes the plant wilt disease while the immobilized form lost this property. On the other hand, the immobilized form achieved great reusability. Thus, exochitinase produced by *A. awamori* EM66 could be useful for application in diverse areas such as biotechnology and agro-industry.
